# Human antigen R promotes angiogenesis of endothelial cells cultured with adipose stem cells derived exosomes via overexpression of vascular endothelial growth factor in vitro

**DOI:** 10.1080/21623945.2021.1982577

**Published:** 2021-10-11

**Authors:** Guo Li, Youbai Chen, Yudi Han, Tian Ma, Yan Han

**Affiliations:** a Department of Plastic and Reconstructive Surgery, First Medical Center of Chinese PLA General Hospital, Beijing, China; b Graduate school, Chinese PLA Medical School, Beijing, China

**Keywords:** Angiogenesis, adipose-derived stem cells, exosomes, human antigen R, human umbilical vein endothelial cells, vascular endothelial growth factor

## Abstract

Recent studies showed that exosomes obtained from adipose-derived stem cells (ADSCs) could improve the angiogenesis of fat grafts via overexpression of vascular endothelial growth factor (VEGF). Human antigen R (HuR) promotes the expression of VEGF in many cancers, but the effect of HuR in normal endothelial cells in the presence of ADSC-derived exosomes remains unclear. We aimed to investigate the effect of HuR on the expression of VEGF and angiogenesis of human umbilical vein endothelial cells (HUVECs) cultured with ADSCs-derived exosomes. The HuR-overexpressed HUVECs (HuR-HUVECs) were cocultured with ADSCs-derived exosomes. qRT-PCR and Western blotting were performed to examine the stability and expression of VEGF-A mRNA and protein. The proliferation, migration, and proangiogenic capacity of HuR-HUVECs were evaluated using cell counting kit-8 (CCK-8), scratch wound healing, and Matrigel tube formation assay. qRT-PCR showed that HuR-HUVECs had higher expression and slower attenuation of VEGF-A mRNA. Western blotting confirmed higher expression of VEGF-A in HuR-HUVECs. CCK-8, scratch wound healing, and Matrigel tube formation assay demonstrated an increased proangiogenic effect in HuR-HUVECs. HuR promotes angiogenesis of HUVECs cocultured with ADSCs-derived exosomes via stabilization and overexpression of VEGF in vitro. The HuR/VEGF pathway is an important regulatory mechanism of angiogenesis in endothelial cells.

## Introduction

Fat grafting has been widely used in plastic, reconstructive, and aesthetic surgery for the treatment of atrophic scars, congenital malformations, facial rejuvenation, and breast reconstruction or augmentation, etc. However, the high risk of complications such as absorption and necrosis of grafted fat substantially compromise the long-term effect of fat grafting [1–4]. These complications are mainly attributed to the ischaemic microenvironment due to inadequate and delayed vascularization during the early stage following fat grafting. Therefore, it is essential to promote angiogenesis and establish an effective blood supply to improve the survival of fat grafts [4,5].

Previous studies [6–9] have demonstrated that adipose-derived stem cells (ADSCs) are able to increase the survival of fat grafts through their paracrine capability of various proangiogenic growth factors such as vascular endothelial growth factor (VEGF), a master regulator of neovascularization and angiogenesis. This paracrine function is primarily accomplished via the release of extracellular vesicles e.g. exosomes, which are the major carriers of VEGF. Our previous study [10] showed that ADSCs-derived exosomes could promote neovascularization, alleviate inflammation and apoptosis in skin flap, thus enhancing flap survival after ischaemia-reperfusion injury in a rat model. The most recent studies by our group [11,12] further found that exosomes derived from hypoxia-preconditioned ADSCs could facilitate angiogenesis and attenuate inflammation, thus improving the survival of fat grafts via VEGF/VEGF-R signalling pathway. However, the key molecular triggers and underlying mechanism of VEGF transcription and expression in the environment of ADSCs-derived exosomes remains unclear.

RNA-binding proteins (RBPs) play a critical role in the regulation of VEGF gene transcription and expression such as VEGF mRNA transportation, localization and translation [13]. As one of the most important RBPs, Human antigen R (HuR) stabilizes VEGF mRNA and facilitates its translation into VEGF by binding to the adenine- and uridine-rich elements in the 3ʹ- untranslated region [14,15]. It is well established that overexpression of HuR promotes VEGF mRNA translation and upregulates VEGF expression in many cancer cells [16–21]. However, the interactions between HuR and VEGF in noncancerous vascular endothelial cells under the circumstance of ADSC-derived exosomes are not well understood. To facilitate angiogenesis and improve the survival of fat grafts, it is essential to understand the effect of HuR on the expression of VEGF in noncancerous vascular endothelial cells.

The purpose of this study was to investigate the effect of HuR on the expression of VEGF and angiogenesis of human umbilical vein endothelial cells (HUVECs) cultured with ADSCs-derived exosomes. Based on the literature and our previous studies, we hypothesized that HuR would promote the angiogenesis of HUVECs via overexpression of VEGF.

## Materials and methods

### Isolation and identification of ADSCs and ADSCs-derived exosomes

After approved by the Ethics Committee of the Chinese PLA General Hospital, we harvested human subcutaneous adipose tissue from healthy females aged from 18 to 30 years by abdominal liposuction. Informed consent was provided by each participant. Human ADSCs were isolated from lipoaspirates and cultured as previously described [22]. To identify ADSCs, surface markers including CD19, CD44, CD90, CD105, CD34, CD45 and CD73 (Abcam, UK) were examined using flow cytometry (BD Accrui C6, USA). In addition, adipogenic, osteogenic and chondrogenic differentiation of ADSCs was induced and verified using Oil red O, Alizarin Red and Alcian Blue staining, respectively.

Isolation and identification of ADSCs-derived exosomes were performed as follows. Conditioned medium of passage-3 ADSCs was collected, centrifuged at 1500 × g for 5 min and 3000 × g for 5 min to remove cell pellets. The supernatant was filtered through a 0.22 μm filter (Millipore, USA), ultracentrifuged at 100,000 × g for 90 min using 45 Ti rotor (Beckman Coulter, USA). The pellets were resuspended with 1 mL PBS and centrifuged at 100,000 × g for 60 min and resuspended in 100 μL PBS for further use. To identify ADSCs-derived exosomes, exosomal surface markers tumour susceptibility gene 101 (TSG 101), CD 63 and Calnexin were detected using Western blotting. Transmission electron microscopy (TEM, HITACHI H-7000FA, Japan) was performed to examine the size and morphology of ADSCs-derived exosomes. The size distribution and concentration of exosomes were analysed by using nanoparticle tracking analysis system (Particle Metrix Zetaview, Germany).

### Lentiviral transfection of HuR genes to HUVECs and verification

HUVECs, purchased from National Infrastructure of Cell Line Resource, were cultured in DMEM supplemented with 10% foetal bovine serum at a concentration of 3 × 10^5^ cells/mL. The medium was replaced with DMEM supplemented with 8 μg/mL polybrene at 70–80% confluency. PBS, Green fluorescence protein (GFP) lentivirus, or GFP-HuR lentivirus (Beijing BGBiotech Co. Ltd., China) were added to HUVECs at a multiplicity of infection (MOI) of 30, respectively. The HuR gene was labelled with a 3× FLAG tag protein gene, which was used as a marker of successful transduction of HuR into HUVECs. The 3 groups of HUVECs were defined as wild type HUVECs (WT-HUVECs), GFP-HUVECs and HuR-HUVECs. Lentiviral transfection was performed using a commercial lentiviral transfection kit according to the manufacturer protocol (BioGeek™ Lentiviral Packaging Kit, Syngentech, China). The medium was replaced with DMEM 24 hours later and supplemented with 0.4 μg/mL puromycin 48 hours later when GFP expression was detected by fluorescence microscopy. The medium was replaced every 2 to 3 days until GFP-positive cells reached 95% confluency or greater.

The 3 groups of HUVECs were lysed by RIPA. The total protein was assessed by the BCA protein assay kit (Thermo Fisher Scientific, USA). The lysates were electrophoresed by using 12% SDS gels and transferred to polyvinylidene fluoride membranes. Each blot was blocked and incubated overnight with the following primary antibodies: Flag tag Rabbit PolyAb or mouse anti-β-actin antibody (1:2000, Sigma-Aldrich, USA). Horseradish peroxidase-conjugated anti-rabbit or anti-mouse IgG (1:2000, ZSGB-BIO, USA) secondary antibody was added at room temperature for 1 hour. The target proteins were visualized with Amersham Hyperfilm ECL (GE Healthcare, USA).

### Expression of VEGF-A mRNA and protein in HUVECs

Quantitative real-time polymerase chain reaction (qRT-PCR) was performed to examine the expression of VEGF-A mRNA. Primers for VEGF-A and 18s rRNA were listed in Table 1. The WT-HUVECs, GFP-HUVECs and HuR-HUVECs were seeded at a density of 2 × 10^5^ cells per well into 6-well plates in DMEM containing 50 μg/mL ADSCs-derived exosomes (exosome-DMEM). This seeding condition was used throughout the following experiments unless otherwise specified. The 3 groups of HUVECs were collected 48 hours later. Total RNA was extracted using Trizol (Tiangen, China) and cDNA was amplified by qRT-PCR according to the manufacturer’s instruction (TaKaRa, Japan). Relative expression of VEGF-A mRNA was analysed by using the 2^−ΔΔCT^ method.

**Table 1.** Primers used for qRT-PCR of target genes

Primers	Forward	Reverse
VEGF-A	5ʹ-TGCCATCCAATCGAGACCC-3’	5ʹ-ATGTTGGACTCCTCAGTGGGC-3’
18s rRNA	5ʹ-GTAACCCGTTGAACCCCATT-3’	5ʹ-CCATCCAATCGGTAGTAGCG-3’

Western blotting was performed as previously described, except the rabbit anti-VEGF-A antibody (1:2000, Abcam, UK) was used as the primary antibody. The semiquantitative densitometric analysis of the bands was performed using TotalLab Quant 11.5 software (Newcastle upon Tyne, UK).

### Stability of VEGF-A mRNA in HUVECs

To inhibit the transcription, 5 μg/mL Actinomycin D (Sigma-Aldrich, USA) was added to the HUVECs 48 hours after seeding. HUVECs were collected at 0, 1, 2 and 4 hours later. The qRT-PCR was performed and the percentage of remaining VEGF-A mRNA at different time points was calculated to evaluate the half-life of VEGF-A mRNA.

### Cell proliferation, migration, and angiogenesis of HUVECs

To investigate the effect of HuR on the proliferation, migration and angiogenesis of HUVECs, cell counting kit-8 assay (CCK-8), scratch wound healing assay, and tube formation assay in Matrigel were performed. Briefly, the 3 groups of HUVECs were seeded at a density of 5 × 10^3^ cells per well with exosome-DMEM. The CCK-8 (Dojindo, Japan) was used to evaluate the cell proliferation. The optical density (OD) was measured at 450 nm by using a microplate reader (Mindray, China).

Scratch wounds were created by using 200 μL pipette tip at a cell confluency of 90%. The medium was replaced by exosome-DMEM. Photos were taken at 0, 12 and 24 hours afterwards. The residual area of the scratch wound was measured by using Image-Pro Plus 6.0 software (Media Cybernetics, USA).

Matrigel (BD Biosciences, USA) was used to assess the tube formation of HUVECs. The 3 groups of HUVECs were seeded at a density of 2 × 10^4^ cells per well into 24-well plates coated by Matrigel, and cultured in exosome-DMEM for 6 and 12 hours. Tube formation was assessed by phase-contrast microscopy (OLYMPUS, Japan) and the total tube length was measured using Image-Pro Plus 6.0 software.

### Statistical analysis

All data were presented as mean ± standard deviations (SD) if they were normally distributed. All experiments were independently repeated three times. Comparison between 2 groups was performed using the student *t* test and comparison among 3 groups was performed using the one-way analysis of variance with Turkey’s HSD post-hoc test. Statistical analysis was performed using IBM SPSS Statistics software 24.0 (IBM corporation, USA). A *P* value <0.05 was considered statistically significant.

## Results

### Characterization of ADSCs and ADSCs-derived exosomes

**Figure 1.**
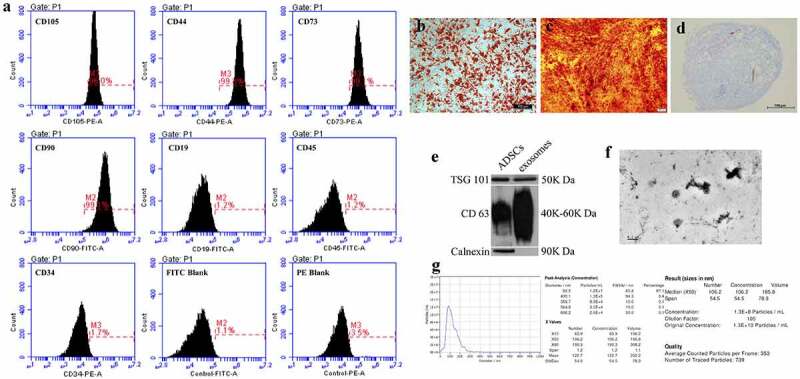
Identification of ADSCs and ADSCs-derived exosomes. (a) Flow cytometry for ADSCs. (b) oil red O staining for adipogenic differentiation. scale bar = 100 μm. (c) alizarin red staining for osteogenic differentiation. scale bar = 200 μm. (d) alcian blue staining for chondrogenic differentiation. scale bar = 100 μm. (e) western blotting for exosomal markers (TSG 101, CD 63 and calnexin). (f) transmission electron microscopy of exosome morphology. SCALE bar = 0.2 μm. (g) nanoparticle tracking analysis

Flow cytometry showed positive expression of CD44, CD73, CD90 and CD105, and negative expression of CD19, CD34 and CD45 of the tested cells (Figure 1(a)). Oil red O, Alizarin Red and Alcian blue staining demonstrated adipogenic (Figure 1(b)), osteogenic (Figure 1(c)) and chondrogenic (Figure 1(d)) differentiation. These results confirmed that the isolated cells were ADSCs [23]. Western blot showed positive expressions of TSG 101 and CD 63, and negative expression of Calnexin (Figure 1(e)) in ADSC-derived exosomes. Morphologically, ADSCs-derived exosomes exhibited a double-sided concave disk shape under TEM (Figure 1(f)). The nanoparticle tracking analysis demonstrated that the median size of ADSCs-derived exosome was 106.2 ± 54.5 nm (ranged from 60.9 nm to 190.3 nm). The concentration of exosomes was 1.3 × 10^10^ particles/ml (Figure 1(g)).

### HuR-overexpressed HUVECs

**Figure 2.**
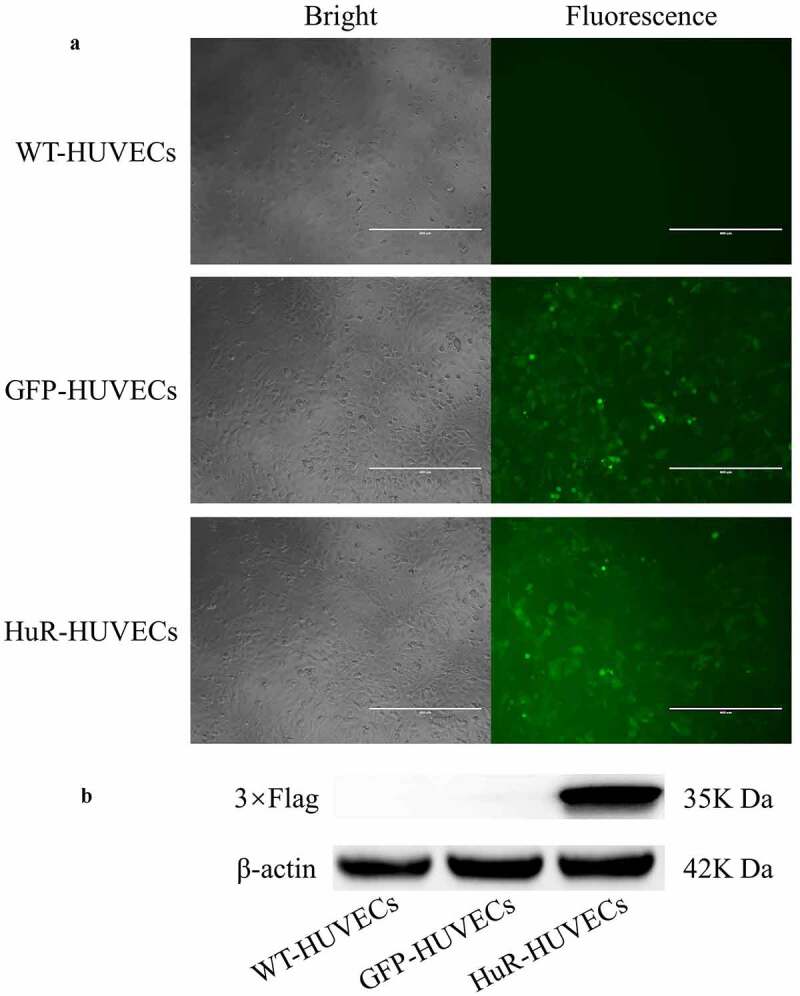
Detection of lentiviral transfection of HuR genes to HUVECs. (a) green fluorescence is observed in GFP-HUVECs and HuR-HUVECs groups. Scale bar = 400 μm. (b) western blotting for 3× FLAG detection shows 3× FLAG is only expressed in HuR-HUVECs

Fluorescence microscopy showed green fluorescence in GFP-HUVECs and HuR-HUVECs (Figure 2(a)), indicating that GFP gene was successfully transfected into HUVECs in both groups. Furthermore, Western blot only detected the expression of 3× FLAG in HuR-HUVECs (Figure 2(b)), indicating a successful transduction of HuR into the HuR-HUVECs.

### The Expressions and Stability of VEGF-A in HuR-HUVECs

**Figure 3.**
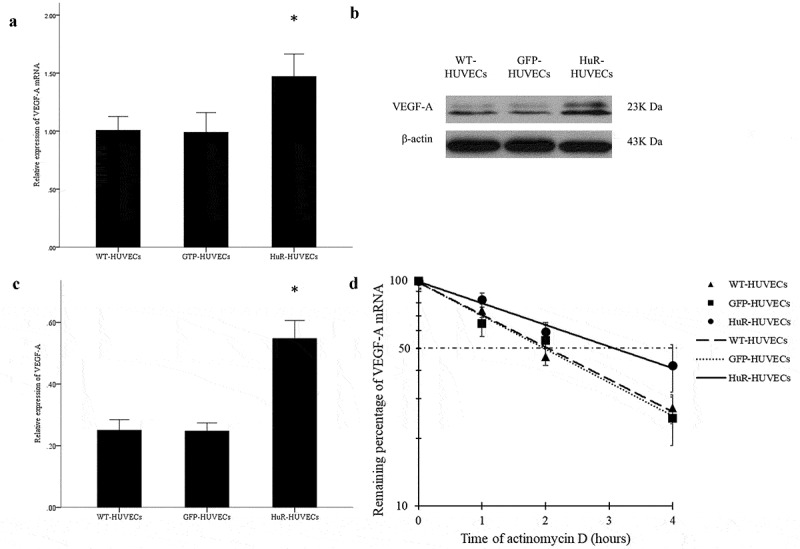
The expression and stability of VEGF-A mRNA is elevated in HuR-HUVECs. (a) quantitative analysis of the relative expression of VEGF-A mRNA. (b) the result of western blot for VEGF-A. (c) quantitative analysis of the relative expression of VEGF-A protein. (d) VEGF-A mRNA has slower decay in HuR-HUVECs compared to the other two groups of HUVECs, indicating an enhanced stability of VEGF-A mRNA. *, *P* < 0.05

qRT-PCR showed an enhanced expression of VEGF-A mRNA in HuR-HUVECs group compared to the other 2 groups (Figure 3(a)). In addition, Western blotting (Figure 3(b)) and semiquantitative analysis (Figure 3(c)) demonstrated a higher expression of VEGF-A in HuR-HUVECs compared to the other 2 groups. qRT-PCR at different time points showed that the half-life of VEGF-A mRNA in HuR-HUVECs, WT-HUVECs, and GFP-HUVECs was 3.10 hours, 2.05 hours and 1.98 hours, respectively (Figure 3(d)). VEGF-A mRNA had slower attenuation in HuR-HUVECs compared to the other 2 groups, indicating an enhanced stability of VEGF mRNA in HuR-HUVECs.

### Cell proliferation, migration, and angiogenesis of HUVECs

**Figure 4.**
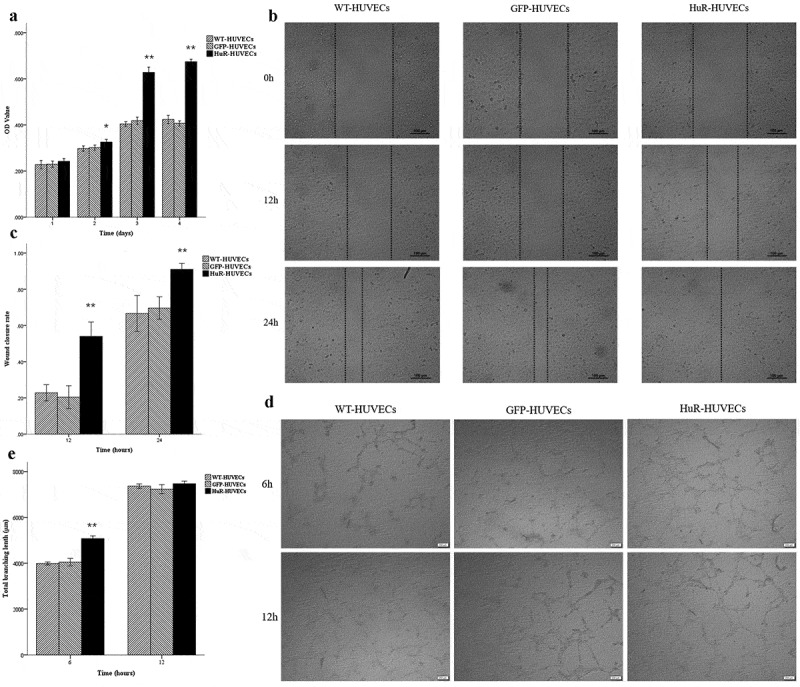
HuR-HUVECs shows increased proangiogenic activities in vitro. (a) CCK-8 cell proliferation assay. (b) scratch wound cell migration assay. Scale bar = 100 μm. (c) quantitative analysis of residual fractional area of the scratch wound. (d) matrigel tube formation assay. scale bar = 200 μm. (e) quantitative analysis of total tube length. *, *P* < 0.05. **, *P* < 0.01

CCK-8 assay showed that the OD values in all 3 groups increased as time passed. Although no significant difference among 3 groups of HUVECs was found on Day 1, HuR-HUVECs exhibited greater OD value than the other 2 groups on Day 2, 3 and 4. The result indicated that HuR promoted the proliferation of HUVECs (Figure 4(a)). Scratch wound healing assay showed that the migration of HuR-HUVECs was significantly increased than the other 2 groups at 12 and 24 hours (Figures 4(b-c)), indicating that HuR enhanced the migration capacity of HUVECs. Matrigel tube formation assay demonstrated a longer total length of tube structure in HuR-HUVECs groups compared to its counterparts at 6 hours, but not at 12 hours. (Figures 4(d-e)), indicating an early enhanced angiogenesis in HuR-HUVECs.

## Discussion

The results of this study have demonstrated that HuR promotes the proliferation, migration, and angiogenesis of HUVECs cocultured with ADSCs-derived exosomes via stabilization and overexpression of VEGF in vitro.

Previous studies have shown that HuR enhanced the stability of VEGF mRNA, overexpression of VEGF, and subsequent angiogenesis in various types of cancer cells. For example, Levy et al. [24] reported that overexpression of HuR increased the stability of VEGF mRNA in clear cell renal cell carcinoma. Xie et al. [25] found that exosomal circSHKBP1 promoted gastric cancer progression via regulating the miR-582-3p/HUR/VEGF axis. Mitsunari et al. [20] noted that HuR was positively associated with malignant aggressiveness of prostate cancer via upregulation of cell proliferation, migration, and VEGF expression. Miyata et al. [26] reported that high expression of HuR was associated with malignant aggressiveness and prognosis in bladder cancer.

However, the role of HuR in VEGF expression and angiogenesis in non-cancer normal endothelial cells remains controversial. Hung et al. noted that HuR modulated VEGF expression in human corneal epithelial cells under hypoxia. Chang et al. [27] reported that HuR promoted VEGF mRNA expression by antagonizing the suppressive effect of miR-200b in bone marrow derived macrophages. Amadio et al. [28] found that Protein kinase C activation affected VEGF expression via HuR in a pericytic/endothelial coculture model. In contrast, Kurosu et al. [19] found overexpression of HuR and consequent upregulation of VEGF in tumour endothelial cells, but not in normal endothelial cells. In accordance with the former, the results of our study corroborate that HuR promotes VEGF expression and angiogenesis of normal endothelial cells cocultured with ADSC-derived exosomes.

Previous studies showed that ADSC-derived exosomes enhanced angiogenesis via upregulation of VEGF expression, but further researches are required to elucidate the interplay between ADSC-derived exosomes and HuR/VEGF pathway. An et al. [29] and Ren et al. [30] reported that exosomes facilitated vascularization of HUVECs by activating the AKT and ERK signalling pathways and upregulating the expression of VEGF. Cho et al. [31] found that exosomes elevated the expression of VEGF via mediation of SMAD2 signalling pathway. Zhu et al. [32] showed that the proangiogenic effect of extracellular vesicles derived from ADSCs in vitro was attributed to the activation of let-7/argonaute 1/VEGF signalling pathway. Our previous studies also validated the proangiogenic effect of ADSCs-derived exosomes, particularly in upregulating VEGF expression under hypoxia condition.

A major limitation of our study is that ADSCs-derived exosomes were used as a coculture model with HUVECs. The interaction between ADSC-derived exosomes and the HuR/VEGF pathway in endothelial cells was beyond the scope of the current study. Therefore, the molecular mechanism of proangiogenic capability of ADSCs-derived exosomes is still unclear. In addition, down-regulation or knockout of HuR was absent. Future animal study will be required to better elucidate the effect of HuR on the angiogenesis and survival of fat grafts. Despite these limitations, this is the first study investigating the effect of HuR on VEGF expression and angiogenesis of HUVECs in the presence of ADSCs-derived exosomes in vitro. The results of this study have corroborated that the proangiogenic function of HuR/VEGF axis in non-cancer normal endothelial cells, which may be used as a target in fat grafts, tissue regeneration and reconstruction.

## Conclusion

This study demonstrates that HuR promotes the proliferation, migration, and angiogenesis of HUVECs cocultured with ADSCs-derived exosomes via stabilization and overexpression of VEGF in vitro. The HuR/VEGF pathway is an important regulatory mechanism of angiogenesis in non-cancer normal endothelial cells.

## References

[1] Condé-Green A, Marano AA, Lee ES, et al. Fat grafting and adipose-derived regenerative cells in burn wound healing and scarring: a systematic review of the literature. Plast Reconstr Surg. 2016;137(1):302–312. 26710034 10.1097/PRS.0000000000001918

[2] Del Vecchio DA, Villanueva NL, Mohan R, et al. Clinical Implications of Gluteal Fat Graft Migration: a Dynamic Anatomical Study. Plast Reconstr Surg. 2018;142(5):1180–1192. 30102666 10.1097/PRS.0000000000005020

[3] Pu LL, Yoshimura K, Coleman SR. Future perspectives of fat grafting. Clin Plast Surg. 2015;42(3):389–x. 26116945 10.1016/j.cps.2015.03.007

[4] Pu LL. Mechanisms of fat graft survival. Ann Plast Surg. 2016;77(1):S84–86. 26808753 10.1097/SAP.0000000000000730

[5] Zhao J, Yi C, Li L, et al. Observations on the survival and neovascularization of fat grafts interchanged between C57BL/6-gfp and C57BL/6 mice. Plast Reconstr Surg. 2012;130(3):398e–406e. 10.1097/PRS.0b013e31825dbfd3 22575853

[6] Yoshimura K, Sato K, Aoi N, et al. Cell-assisted lipotransfer for facial lipoatrophy: efficacy of clinical use of adipose-derived stem cells. Dermatol Surg. 2008;34(9):1178–1185. 18513295 10.1111/j.1524-4725.2008.34256.x

[7] Yoshimura K, Sato K, Aoi N, et al. Cell-assisted lipotransfer for cosmetic breast augmentation: supportive use of adipose-derived stem/stromal cells. Aesthetic Plast Surg. 2008;32(1):48–55. discussion 56-47. 17763894 10.1007/s00266-007-9019-4 PMC2175019

[8] Laloze J, Varin A, Gilhodes J, et al. Cell-assisted lipotransfer: friend or foe in fat grafting? Systematic review and meta-analysis. J Tissue Eng Regen Med. 2018;12(2):e1237–e1250. 28719946 10.1002/term.2524

[9] Suga H, Glotzbach JP, Sorkin M, et al. Paracrine mechanism of angiogenesis in adipose-derived stem cell transplantation. Ann Plast Surg. 2014;72(2):234–241. 23636112 10.1097/SAP.0b013e318264fd6a PMC3740067

[10] Bai Y, Han YD, Yan XL, et al. Adipose mesenchymal stem cell-derived exosomes stimulated by hydrogen peroxide enhanced skin flap recovery in ischemia-reperfusion injury. Biochem Biophys Res Commun. 2018;500(2):310–317. 29654765 10.1016/j.bbrc.2018.04.065

[11] Han YD, Bai Y, Yan XL, et al. Co-transplantation of exosomes derived from hypoxia-preconditioned adipose mesenchymal stem cells promotes neovascularization and graft survival in fat grafting. Biochem Biophys Res Commun. 2018;497(1):305–312. 29428734 10.1016/j.bbrc.2018.02.076

[12] Han Y, Ren J, Bai Y, et al. Exosomes from hypoxia-treated human adipose-derived mesenchymal stem cells enhance angiogenesis though VEGF/VEGF-R. Int J Biochem Cell Biol. 2019;109:59–68. 30710751 10.1016/j.biocel.2019.01.017

[13] Wang Y, Li Y, Toth JI, et al. N6-methyladenosine modification destabilizes developmental regulators in embryonic stem cells. Nat Cell Biol. 2014;16(2):191–198. 24394384 10.1038/ncb2902 PMC4640932

[14] Ma WJ, Cheng S, Campbell C, et al. Cloning and characterization of HuR, a ubiquitously expressed Elav-like protein. J Biol Chem. 1996;271(14):8144–8151. 8626503 10.1074/jbc.271.14.8144

[15] Hinman MN, Lou H. Diverse molecular functions of Hu proteins. Cell Mol Life Sci. 2008;65(20):3168–3181. 18581050 10.1007/s00018-008-8252-6 PMC2580827

[16] Sakuma T, Nakagawa T, Ido K, et al. Expression of vascular endothelial growth factor-A and mRNA stability factor HuR in human meningiomas. J Neurooncol. 2008;88(2):143–155. 18317686 10.1007/s11060-008-9559-8

[17] Datta K, Mondal S, Sinha S, et al. Role of elongin-binding domain of von hippel lindau gene product on HuR-mediated VPF/VEGF mRNA stability in renal cell carcinoma. Oncogene. 2005;24(53):7850–7858. 16170373 10.1038/sj.onc.1208912

[18] Ido K, Nakagawa T, Sakuma T, et al. Expression of vascular endothelial growth factor-A and mRNA stability factor HuR in human astrocytic tumors. Neuropathology. 2008;28(6):604–611. 18498284 10.1111/j.1440-1789.2008.00926.x

[19] Kurosu T, Ohga N, Hida Y, et al. HuR keeps an angiogenic switch on by stabilising mRNA of VEGF and COX-2 in tumour endothelium. Br J Cancer. 2011;104(5):819–829. 21285980 10.1038/bjc.2011.20 PMC3048211

[20] Mitsunari K, Miyata Y, Asai A, et al. Human antigen R is positively associated with malignant aggressiveness via upregulation of cell proliferation, migration, and vascular endothelial growth factors and cyclooxygenase-2 in prostate cancer. Transl Res. 2016;175:116–128. 27140699 10.1016/j.trsl.2016.04.002

[21] Osera C, Martindale JL, Amadio M, et al. Induction of VEGFA mRNA translation by CoCl2mediated by HuR. RNA Biol. 2015;12(10):1121–1130. 26325091 10.1080/15476286.2015.1085276 PMC4829335

[22] Zuk PA, Zhu M, Mizuno H, et al. Multilineage cells from human adipose tissue: implications for cell-based therapies. Tissue Eng. 2001;7(2):211–228. 11304456 10.1089/107632701300062859

[23] Dominici M, Le Blanc K, Mueller I, et al. Minimal criteria for defining multipotent mesenchymal stromal cells. the international society for cellular therapy position statement. Cytotherapy. 2006;8(4):315–317. 16923606 10.1080/14653240600855905

[24] Levy NS, Chung S, Furneaux H, et al. Hypoxic stabilization of vascular endothelial growth factor mRNA by the RNA-binding protein HuR. J Biol Chem. 1998;273(11):6417–6423. 9497373 10.1074/jbc.273.11.6417

[25] Xie M, Yu T, Jing X, et al. Exosomal circSHKBP1 promotes gastric cancer progression via regulating the miR-582-3p/HUR/VEGF axis and suppressing HSP90 degradation. Mol Cancer. 2020;19(1):112. 32600329 10.1186/s12943-020-01208-3 PMC7322843

[26] Miyata Y, Watanabe S, Sagara Y, et al. High expression of HuR in cytoplasm, but not nuclei, is associated with malignant aggressiveness and prognosis in bladder cancer. PLoS One. 2013;8(3):e59095. 23516604 10.1371/journal.pone.0059095 PMC3596286

[27] Chang SH, Lu YC, Li X, et al. Antagonistic function of the RNA-binding protein HuR and miR-200b in post-transcriptional regulation of vascular endothelial growth factor-A expression and angiogenesis. J Biol Chem. 2013;288(7):4908–4921. 23223443 10.1074/jbc.M112.423871 PMC3576095

[28] Amadio M, Osera C, Lupo G, et al. Protein kinase C activation affects, via the mRNA-binding Hu-antigen R/ELAV protein, vascular endothelial growth factor expression in a pericytic/endothelial coculture model. Mol Vis. 2012;18:2153–2164. 22879735 PMC3415319

[29] An Y, Zhao J, Nie F, et al. Exosomes from Adipose-Derived Stem Cells (ADSCs) Overexpressing miR-21 Promote Vascularization of Endothelial Cells. Sci Rep. 2019;9(1):12861. 31492946 10.1038/s41598-019-49339-y PMC6731308

[30] Ren S, Chen J, Duscher D, et al. Microvesicles from human adipose stem cells promote wound healing by optimizing cellular functions via AKT and ERK signaling pathways. Stem Cell Res Ther. 2019;10(1):47. 30704535 10.1186/s13287-019-1152-x PMC6357421

[31] Cho JA, Park H, Lim EH, et al. Exosomes from breast cancer cells can convert adipose tissue-derived mesenchymal stem cells into myofibroblast-like cells. Int J Oncol. 2012;40(1):130–138. 21904773 10.3892/ijo.2011.1193

[32] Zhu Y, Zhang J, Hu X, et al. Extracellular vesicles derived from human adipose-derived stem cells promote the exogenous angiogenesis of fat grafts via the let-7/AGO1/VEGF signalling pathway. Sci Rep. 2020;10(1):5313. 32210269 10.1038/s41598-020-62140-6 PMC7093512

